# Quantitative agreement between AMYclz and CortexID for ^18^F-florbetapir amyloid PET: a retrospective study of 103 patients

**DOI:** 10.1007/s11604-026-02026-1

**Published:** 2026-06-08

**Authors:** Yoichi Otomi, Takayoshi Shinya, Hideki Otsuka, Yushi Kamei, Manduukhai Badarchin, Tomoki Matsushita, Yukiko Takaoka, Yuka Hiroshima, Tomoyasu Matsubara, Koji Fujita, Yukiko Tomioka, Masahito Nakataki, Shusuke Numata, Yuishin Izumi, Masafumi Harada

**Affiliations:** Tokushima University Hospital, Tokushima, Japan

**Keywords:** Amyloid PET, SUVr, Centiloid scale, Alzheimer’s disease, Quantification

## Abstract

**Purpose:**

Quantitative analysis of amyloid PET images is increasingly used to support visual interpretation and to provide objective measures of amyloid burden. However, quantitative values may vary depending on the analysis software used. This study aimed to evaluate the quantitative agreement between two analysis platforms, AMYclz and CortexID, for ^18^F-florbetapir amyloid PET.

**Materials and methods:**

This retrospective study included 103 consecutive patients who underwent ^18^F-florbetapir PET for evaluation of cognitive impairment. Visual interpretation was used to classify scans as amyloid-positive or amyloid-negative. Quantitative analysis was performed using CortexID and AMYclz software to obtain global cortical standardized uptake value ratios (SUVr). AMYclz additionally provided Centiloid scale (CL) values. The ability of quantitative metrics to differentiate visually positive and negative scans was evaluated using receiver operating characteristic (ROC) analysis, and agreement between software platforms was assessed using correlation, linear regression, and Bland–Altman analysis.

**Results:**

Among 103 patients, 59 were visually classified as amyloid-positive and 44 as amyloid-negative. CortexID SUVr values were significantly higher in amyloid-positive patients than in amyloid-negative patients (1.30 ± 0.14 vs. 0.97 ± 0.09, *p* < 0.001). AMYclz SUVr showed similar separation between groups (1.34 ± 0.14 vs. 0.99 ± 0.09, *p* < 0.001). ROC analysis demonstrated excellent discrimination for CortexID SUVr (AUC = 0.986), AMYclz SUVr (AUC = 0.996), and CL values (AUC = 0.996). CortexID and AMYclz SUVr values showed strong correlation (*r* = 0.957) with minimal systematic bias. Discordant classification between the two software platforms was observed in three cases (2.9%), all near the diagnostic threshold.

**Conclusion:**

AMYclz and CortexID demonstrated excellent quantitative agreement for ^18^F-florbetapir amyloid PET. Both AMYclz SUVr and CL values showed excellent ability to differentiate visually amyloid-positive and amyloid-negative scans, supporting the reliability of quantitative amyloid PET analysis across different software platforms in clinical practice.

## Introduction

Amyloid positron emission tomography (PET) has become an important imaging tool for the evaluation of Alzheimer’s disease (AD) and other causes of cognitive impairment [1]. Visual interpretation remains the standard method for clinical assessment of amyloid PET scans; however, quantitative analysis has increasingly been used to support interpretation, particularly in borderline cases or for research applications [2].

Recent advances in biomarker research have further changed the conceptual framework of AD diagnosis. The 2024 revised diagnostic criteria proposed by the National Institute on Aging and the Alzheimer’s Association define AD primarily as a biological disease characterized by abnormal biomarkers such as amyloid PET, cerebrospinal fluid markers, or plasma biomarkers [3]. These criteria emphasize that AD pathology can be detected in vivo even before the onset of clinical symptoms. However, this biomarker-based definition has also generated ongoing discussion regarding the role of clinical symptoms in the diagnosis of AD [4].

In parallel with these conceptual developments, the clinical use of amyloid PET has expanded. Appropriate use criteria for amyloid PET imaging were originally proposed in 2013 to guide its clinical application in patients with cognitive impairment [5], and these recommendations have recently been updated to reflect advances in biomarker-based diagnostic frameworks and the emergence of disease-modifying therapies for AD [6].

Several software platforms, including CortexID and AMYclz, have been developed to provide automated quantification of amyloid PET images [7–10]. These tools typically calculate standardized uptake value ratios (SUVr) using predefined cortical regions and reference regions. Quantitative metrics are increasingly used to support visual interpretation and to improve reproducibility of amyloid PET assessment [11]. AMYclz has increasingly been adopted in clinical practice in Japan, highlighting the need for validation against established analysis platforms. However, quantitative values may vary depending on the analysis software and processing pipeline, and the agreement between different software platforms remains an important issue for clinical implementation. Validation of quantitative consistency in routine clinical settings remains important, even for software that has received regulatory approval.

The aim of the present study was therefore to evaluate the quantitative agreement between two analysis platforms, CortexID and AMYclz, for ^18^F-florbetapir amyloid PET in a clinical cohort. In addition to SUVr measurements, AMYclz provides Centiloid scale (CL) values, a standardized scale designed to harmonize quantitative amyloid PET measurements across tracers, analysis pipelines, and imaging centers [2]. Because different institutions use different quantitative software platforms, understanding the agreement between commonly used analysis tools is important for the consistent interpretation of amyloid PET in clinical practice and for harmonization of quantitative amyloid PET assessment across institutions.

## Materials and methods

### Study population

This retrospective study included consecutive patients who underwent brain amyloid PET with ^18^F-florbetapir at our institution between August 2024 and February 2026. Patients were referred for evaluation of cognitive impairment or suspected Alzheimer’s disease. A total of 103 patients were included in the analysis. Visual classification of amyloid status was based on clinical PET reports from routine clinical practice. Scans were categorized as amyloid-positive or amyloid-negative based on standard criteria. Visual interpretation was performed independently by two experienced nuclear medicine physicians in accordance with established visual assessment criteria. Although CL values were available as part of routine clinical practice, the final classification was based primarily on visual assessment rather than predefined quantitative thresholds. Discrepancies were resolved by consensus. Baseline Mini-Mental State Examination-Japanese (MMSE-J) and Hasegawa Dementia Scale-Revised (HDS-R) scores obtained during routine clinical assessment or referral evaluation around the time of amyloid PET examination were retrospectively reviewed when available. This retrospective study was approved by the Ethics Committee of Tokushima University Hospital (Approval No. 4588), and the requirement for written informed consent was waived.

### PET acquisition

All PET examinations were performed after intravenous administration of 370 MBq of ^18^F-florbetapir. PET imaging was initiated approximately 50 min after tracer injection and acquired for 20 min in three-dimensional mode using a Discovery 710/128 scanner (GE Healthcare, Chicago, IL, USA). Images were reconstructed using a block-sequential regularized expectation maximization algorithm (Q.Clear; GE Healthcare, Chicago, IL, USA) with time-of-flight information and standard corrections for attenuation and scatter. Both AMYclz and CortexID analyses were performed using the same reconstructed images.

### Quantitative analysis

Quantitative analysis was performed using two commercially available software packages:

- CortexID (GE Healthcare, Chicago, IL, USA).
- AMYclz Neuro (PDR Pharma, Tokyo, Japan).

For both software platforms, anatomical standardization was performed using PET and CT images acquired during PET/CT imaging. A global cortical composite standardized uptake value ratio (SUVr) was calculated using predefined cortical regions of interest automatically generated by each software platform. These cortical composite regions included frontal, temporal, parietal, and posterior cingulate/precuneus regions. The cerebellar cortex was used as the reference region according to the default analysis settings for ^18^F-florbetapir PET in each software package.

AMYclz processing was based on spatial normalization using SPM12 to Montreal Neurological Institute (MNI) space and automatically provided CL values based on the standardized Centiloid transformation [8, 9]. CortexID processing also involved automated spatial normalization to standard brain templates and predefined cortical region analysis according to the software pipeline [10]. The two software platforms use different proprietary processing pipelines for anatomical standardization and cortical composite region analysis.

### Statistical analysis

Continuous variables are presented as mean ± standard deviation. Differences in quantitative values between amyloid-positive and amyloid-negative groups were assessed using Student’s t-test. Diagnostic performance of quantitative measures was evaluated using receiver operating characteristic (ROC) analysis, with visual interpretation serving as the reference standard. The area under the ROC curve (AUC) was calculated for CortexID SUVr, AMYclz SUVr, and CL values. Optimal cutoff values were determined using the Youden index. Agreement between CortexID and AMYclz SUVr values was assessed using Pearson correlation analysis, linear regression, and Bland–Altman analysis. All statistical analyses were performed using SPSS (version 29; IBM Corp., Armonk, NY, USA) and R software (version 4.5.3; R Foundation for Statistical Computing, Vienna, Austria). ROC and Bland–Altman analyses were performed using R.

## Results

### Patient characteristics

A total of 103 consecutive patients who underwent ^18^F-florbetapir PET were included in this study. Visual assessment classified 59 patients as amyloid-positive and 44 as amyloid-negative. The mean age was comparable between groups (positive: 76.6 ± 6.5 years; negative: 76.5 ± 7.9 years). Sex distribution was balanced between groups (negative: 22 female / 22 male; positive: 36 female / 23 male). Baseline cognitive assessment scores, including MMSE-J and HDS-R, are summarized in Table 1.

**Table 1:** Patient Characteristics

Variable	All Patients (*n* = 103)	Amyloid-negative (*n* = 44)	Amyloid-positive (*n* = 59)
Age, years (mean ± SD)	76.6 ± 7.1	76.5 ± 7.9	76.6 ± 6.5
Sex (Female/Male)	58 / 45	22 / 22	36 / 23
MMSE-J	23.9 ± 2.8	24.5 ± 2.4	23.5 ± 2.4
HDS-R	20.6 ± 4.1 (*n* = 100)	21.5 ± 4.4	19.8 ± 3.8
CortexID SUVr (mean ± SD)	1.16 ± 0.20	0.97 ± 0.09	1.30 ± 0.14
AMYclz SUVr (mean ± SD)	1.19 ± 0.21	0.99 ± 0.09	1.34 ± 0.14
Centiloid scale (CL) values (mean ± SD)	26.7 ± 37.6	−8.9 ± 15.3	53.2 ± 25.3

### Quantitative differences between amyloid-positive and -negative groups

CortexID SUVr values were significantly higher in amyloid-positive patients than in amyloid-negative patients (1.30 ± 0.14 vs. 0.97 ± 0.09, *p* < 0.001). Similarly, AMYclz SUVr values were significantly higher in the positive group (1.34 ± 0.14 vs. 0.99 ± 0.09, *p* < 0.001). CL values were also significantly higher in amyloid-positive patients than in amyloid-negative patients (53.2 ± 25.3 vs. −8.9 ± 15.3, *p* < 0.001).

### Diagnostic performance

**Fig. 1:**
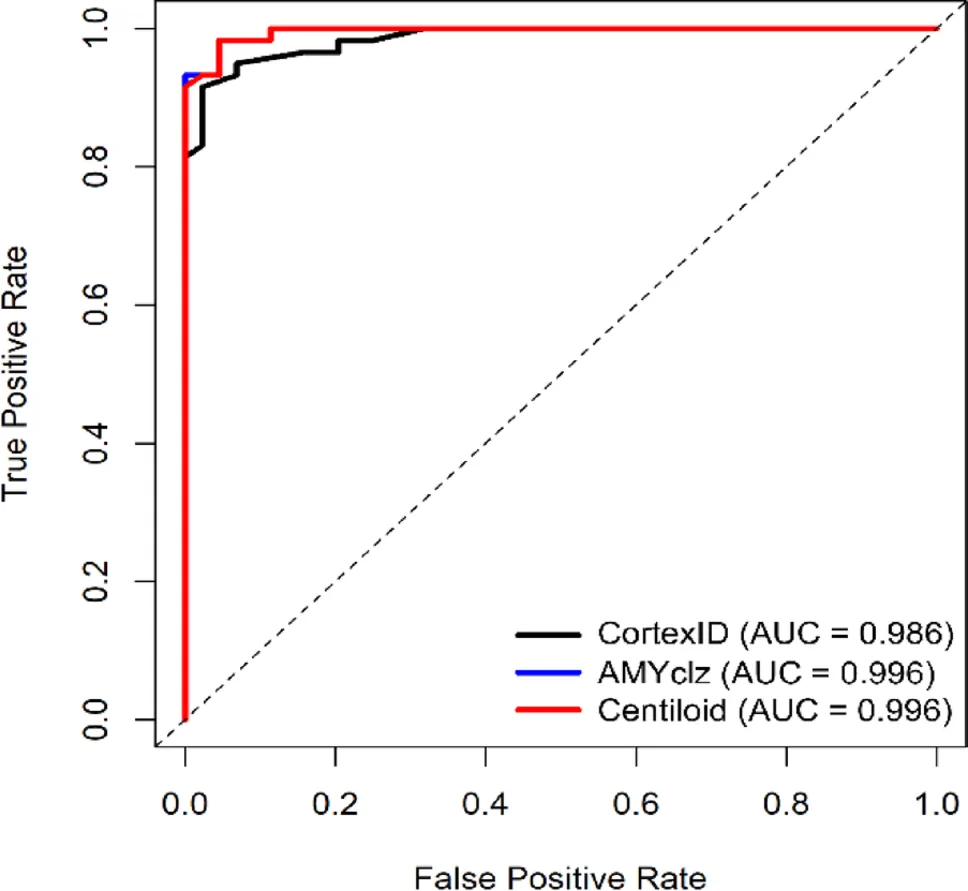
Receiver operating characteristic (ROC) curves for quantitative metrics in ^18^F-florbetapir PET. ROC analysis demonstrated excellent discrimination for CortexID SUVr (AUC = 0.986), AMYclz SUVr (AUC = 0.996), and Centiloid scale (CL) values (AUC = 0.996) for discrimination between visually amyloid-positive and amyloid-negative scans

Receiver operating characteristic (ROC) analysis demonstrated excellent diagnostic performance for all quantitative metrics, with AUC values ranging from 0.986 to 0.996 (Fig. 1). For CortexID SUVr, the area under the curve (AUC) was 0.986 with an optimal cutoff of 1.11, yielding a sensitivity of 0.915 and specificity of 0.977. For AMYclz SUVr, the AUC was 0.996 with an optimal cutoff of 1.15, yielding a sensitivity of 0.983 and specificity of 0.955. For CL values, the AUC was also 0.996 with an optimal cutoff of 19.7, yielding the same sensitivity (0.983) and specificity (0.955). Because CL values are derived from a linear transformation of SUVr, the ROC performance metrics for AMYclz SUVr and CL values were identical. AMYclz SUVr- and CL-based classifications were fully concordant at their respective optimal thresholds.

### Agreement between software platforms

**Fig. 2:**
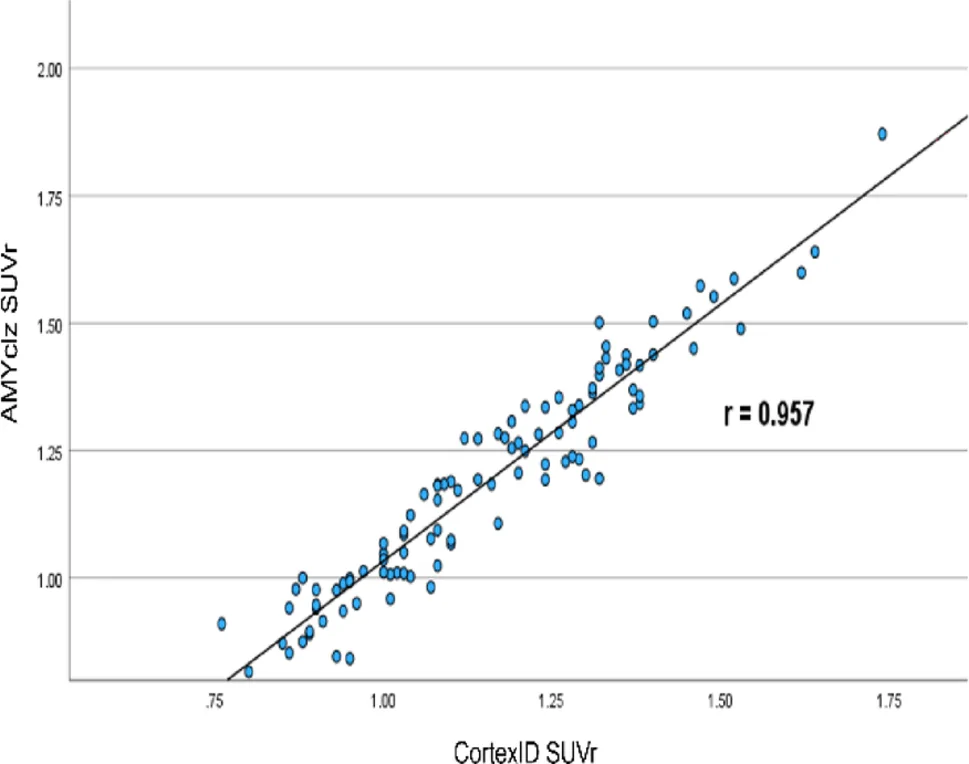
Scatter plot showing the relationship between CortexID SUVr and AMYclz SUVr. A strong linear correlation was observed between the two quantitative measures (*r* = 0.957). The regression line represents the relationship: AMYclz SUVr = 1.007 × CortexID SUVr + 0.027

**Fig. 3:**
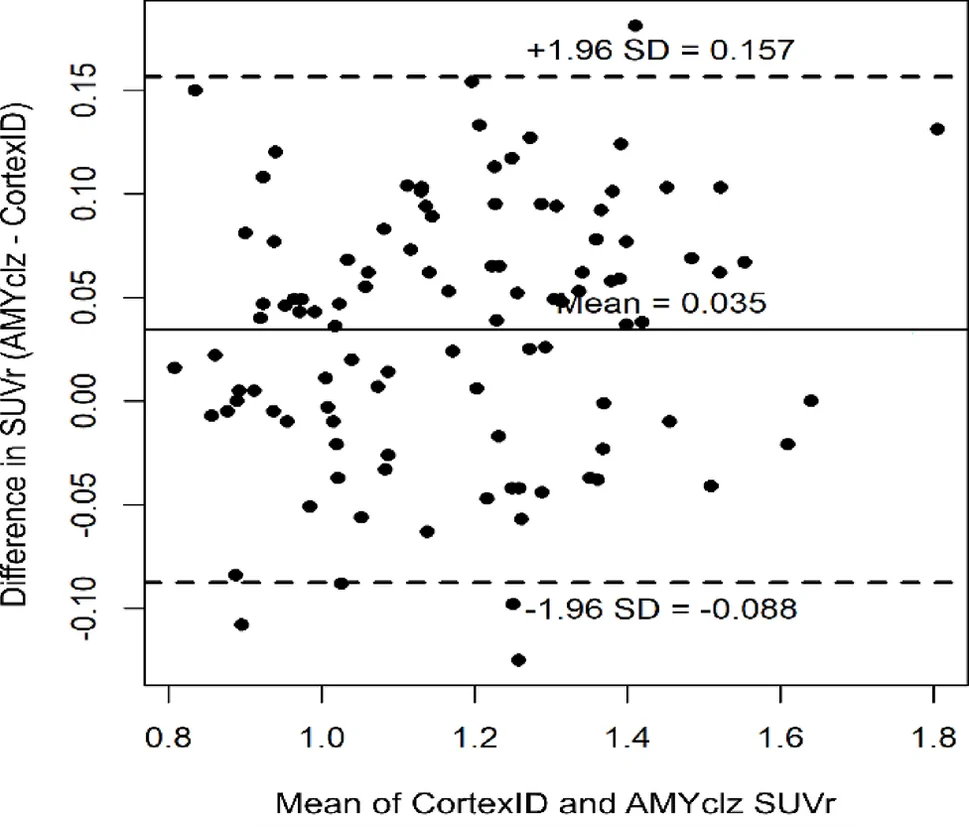
Bland–Altman plot showing agreement between CortexID SUVr and AMYclz SUVr. The plot illustrates the difference between AMYclz and CortexID SUVr values against their mean for each case. The solid line indicates the mean difference, and dashed lines represent the limits of agreement (mean difference ± 1.96 SD)

CortexID and AMYclz SUVr values demonstrated strong linear correlation (*r* = 0.957) (Fig. 2). Linear regression analysis yielded the following relationship: AMYclz SUVr = 1.007 × CortexID SUVr + 0.027. The small mean difference between AMYclz and CortexID SUVr values indicated minimal systematic bias between the two software platforms (Fig. 3).

### Discordant cases

At the software-specific optimal thresholds derived from ROC analysis, discordant classification between CortexID and AMYclz was observed in three cases (2.9%). All discordant cases were located near their respective cutoff thresholds, indicating borderline amyloid burden.

## Discussion

In this study, we evaluated the agreement between two quantitative analysis platforms, CortexID and AMYclz, for ^18^F-florbetapir amyloid PET in a clinical cohort of 103 patients. The results demonstrated excellent concordance between the two software packages. Both AMYclz SUVr and CortexID SUVr showed strong correlation, with minimal systematic bias.

Discordant classification between the two software platforms was observed in only 3 of 103 cases (2.9%), corresponding to a concordance rate of 97.1%, and all discordant cases were located near the diagnostic threshold. In addition, quantitative measures derived from AMYclz, including both SUVr and CL values, showed excellent ability to discriminate between visually amyloid-positive and amyloid-negative scans, consistent with previous reports describing the performance of florbetapir PET imaging [11]. These findings suggest that quantitative assessment using AMYclz provides results highly consistent with those obtained using CortexID and supports the reliability of quantitative amyloid PET analysis across different software platforms. This study therefore further supports the clinical applicability of AMYclz in comparison with a widely used quantitative analysis platform.

Previous studies have investigated the performance of quantitative software tools for amyloid PET analysis and have generally reported strong agreement between different analytical platforms [2, 7, 12, 13]. Several reports comparing quantitative methods for amyloid PET have demonstrated that global cortical SUVr values derived from different software packages show high correlation despite differences in segmentation algorithms and reference regions [2, 7]. Our findings are consistent with these previous observations, demonstrating strong agreement between CortexID and AMYclz SUVr measurements. The high correlation observed in the present study (*r* = 0.957) indicates that quantitative estimates of amyloid burden are largely reproducible across analysis platforms when standardized acquisition protocols are used.

In addition to SUVr measurements, AMYclz provides CL values, which were originally proposed to standardize quantitative amyloid PET measurements across tracers, analysis pipelines, and imaging centers [2]. In the present study, CL values demonstrated performance nearly identical to that of AMYclz SUVr, with the same sensitivity and specificity at the optimal threshold. This finding is expected because CL values are derived from standardized transformations of SUVr measurements; however, the results confirm that CL-based quantification remains consistent with conventional SUVr analysis in routine clinical datasets. The availability of CL values may therefore facilitate harmonization of quantitative amyloid PET results across institutions and software platforms. Rather than demonstrating superiority of one platform, the present findings highlight that both AMYclz and CortexID offer reliable quantitative assessments, with differences that may reflect variations in software design and implementation, such as the availability of CL values in AMYclz. These findings support the practical interchangeability of quantitative measurements across different software environments in routine clinical settings.

Although both software platforms perform automated cortical quantification using the cerebellar cortex as the reference region, differences in atlas construction, spatial normalization strategies, cortical composite region definitions, and image-processing pipelines may contribute to small variations in SUVr values between the two systems. Differences in image processing procedures, such as spatial normalization and smoothing, may also influence quantitative results. Partial volume effects related to cortical atrophy may also influence quantitative SUVr measurements in amyloid PET, although such effects are expected to affect both software platforms similarly in the present study. Despite these methodological differences, the present study demonstrated excellent agreement between CortexID and AMYclz, suggesting that quantitative estimates of amyloid burden are robust across different analysis platforms when standardized acquisition protocols are used.

Reliable quantification of amyloid burden is increasingly important in clinical practice, particularly with the expanding use of disease-modifying therapies for Alzheimer’s disease [14]. The present cohort generally represented patients with mild cognitive impairment to mild dementia severity based on routine cognitive assessments. Quantitative PET metrics may provide objective support for longitudinal assessment and for interpretation of borderline cases in clinical practice. Our findings demonstrate that quantitative measurements derived from AMYclz show excellent agreement with those obtained using CortexID, a widely used analysis platform. This suggests that quantitative interpretation of florbetapir PET can be performed consistently across different software environments, supporting the clinical applicability of quantitative amyloid PET analysis in routine practice. The discordant cases observed in this study were all located near the diagnostic threshold, suggesting that quantitative values may be particularly informative in borderline cases. In clinical practice, quantitative metrics such as SUVr or CL values may provide additional support for visual interpretation when amyloid burden is close to the positivity threshold. These findings highlight the potential role of quantitative analysis as a complementary tool for improving diagnostic confidence in equivocal amyloid PET scans. The high level of agreement observed in this study also suggests that quantitative amyloid PET measurements may remain reproducible across different institutions when standardized imaging protocols and analysis procedures are applied. Such inter-platform consistency may be particularly important for multicenter studies, longitudinal patient follow-up, and future development of quantitative imaging biomarkers that may involve different software environments across institutions. As anti-amyloid therapies become increasingly implemented in clinical practice, reproducible quantitative assessment may become more important not only for baseline evaluation but also for monitoring longitudinal changes in amyloid burden.

Although overall agreement between quantitative measures was high, a small number of cases showed discrepancies between software outputs. Such discrepancies may be attributable to several factors, including differences in image processing algorithms, segmentation accuracy, and region-of-interest definition. In particular, cortical atrophy may affect segmentation performance and lead to variability in quantitative values. In addition, differences in reference region selection and normalization methods across software platforms may contribute to variability in SUVr measurements. While these discrepancies were infrequent in our cohort, they highlight the need for cautious interpretation in selected cases. However, the precise causes of these discrepancies could not be determined in the present study.

Awareness of these sources of variability is particularly important in cases with borderline quantitative values. In such cases, cross-checking with visual assessment and reviewing regional uptake patterns may be helpful in avoiding potential misclassification. Previous comparative studies have also suggested that inter-software variability may influence interpretation in borderline cases and longitudinal evaluations, supporting the importance of understanding platform-specific quantitative characteristics [15–18]. In addition, modest systematic differences among commercially available software platforms have also been reported, potentially reflecting differences in processing pipelines, target region definitions, and anatomical standardization methods [15–18].

Although MRI-based anatomical normalization remains the internationally standardized approach for Centiloid quantification proposed by the GAAIN framework, CT-based workflows are increasingly used in routine PET/CT clinical practice because standardized contemporaneous MRI data suitable for quantitative processing may not always be available in all patients [9]. Previous studies have suggested that CT-based amyloid PET quantification may provide acceptable agreement with MRI-based approaches in clinical settings, although slight underestimation compared with MRI-based normalization has also been reported [9]. Therefore, the present study was designed to evaluate inter-platform agreement under real-world PET/CT conditions using routinely available CT-based analysis workflows. The findings suggest that quantitative amyloid PET measurements may remain highly reproducible across different software platforms even in routine clinical PET/CT settings.

Several limitations should be acknowledged. First, this was a retrospective single-center study, which may limit the generalizability of the findings. Second, the reference standard was based on visual interpretation rather than histopathologic confirmation. Although visual assessment was performed according to established criteria, the potential influence of quantitative information on visual interpretation cannot be completely excluded. Quantitative values, including CL, were available in routine clinical practice; therefore, complete independence of visual interpretation from quantitative information could not be guaranteed. Third, only ^18^F-florbetapir PET was evaluated in the present study, and the results may not necessarily be generalizable to other amyloid PET tracers. Future studies including larger cohorts and different analysis settings may further clarify the robustness of quantitative software comparisons.

## Conclusion

In conclusion, quantitative measurements obtained using AMYclz demonstrated excellent agreement with those derived from CortexID for ^18^F-florbetapir amyloid PET in a cohort of 103 patients. Both AMYclz SUVr and CL values showed excellent ability to differentiate between visually amyloid-positive and amyloid-negative scans. These findings support the reliability and clinical applicability of quantitative amyloid PET analysis across different software platforms.

## Data Availability

The datasets used and/or analyzed during the current study are available from the corresponding author on reasonable request.
